# Evidence for Co-evolution of West Nile Virus and House Sparrows in North America

**DOI:** 10.1371/journal.pntd.0003262

**Published:** 2014-10-30

**Authors:** Nisha K. Duggal, Angela Bosco-Lauth, Richard A. Bowen, Sarah S. Wheeler, William K. Reisen, Todd A. Felix, Brian R. Mann, Hannah Romo, Daniele M. Swetnam, Alan D. T. Barrett, Aaron C. Brault

**Affiliations:** 1 Division of Vector-Borne Diseases, Centers for Disease Control and Prevention, Fort Collins, Colorado, United States of America; 2 Department of Biomedical Sciences, Colorado State University, Fort Collins, Colorado, United States of America; 3 Center for Vectorborne Diseases, University of California, Davis, Davis, California, United States of America; 4 United States Department of Agriculture, Lakewood, Colorado, United States of America; 5 Departments of Pathology and Microbiology & Immunology, University of Texas Medical Branch, Galveston, Texas, United States of America; University of Texas Medical Branch, United States of America

## Abstract

West Nile virus (WNV) has been maintained in North America in enzootic cycles between mosquitoes and birds since it was first described in North America in 1999. House sparrows (HOSPs; *Passer domesticus*) are a highly competent host for WNV that have contributed to the rapid spread of WNV across the U.S.; however, their competence has been evaluated primarily using an early WNV strain (NY99) that is no longer circulating. Herein, we report that the competence of wild HOSPs for the NY99 strain has decreased significantly over time, suggesting that HOSPs may have developed resistance to this early WNV strain. Moreover, recently isolated WNV strains generate higher peak viremias and mortality in contemporary HOSPs compared to NY99. These data indicate that opposing selective pressures in both the virus and avian host have resulted in a net increase in the level of host competence of North American HOSPs for currently circulating WNV strains.

## Author Summary

West Nile virus (WNV) emerged in North America in 1999 and rapidly spread across the U.S. due to the presence of highly susceptible mosquito vectors and avian hosts. One of the major avian reservoirs for WNV in the U.S. is the house sparrow (HOSP), which has low mortality during WNV infection. Here, we investigate how the response of wild HOSPs to WNV infection has changed as a result of the 15-year history of WNV circulation in the U.S. In addition, we evaluated the impact of WNV evolution on viral infection in HOSPs and report that WNV has become increasingly pathogenic to HOSPs over time, while HOSPs may have developed resistance to early WNV strains. Thus, HOSPs are still likely to be an important avian reservoir for WNV in the U.S., and WNV has adapted to its avian hosts during emergence in North America.

## Introduction

West Nile virus (WNV; *Flaviviridae*) is an arbovirus that was first reported in North America in 1999 in New York. By 2003, the virus had spread to the West Coast. WNV has remained endemic in the U.S. due to the high prevalence of competent *Culex* spp. mosquito vectors and avian hosts [1]–[4]. The birds considered to be the most important WNV reservoirs are passerines, which are highly susceptible and maintain high viremias for several days during infection [1], [5]. Because infection of *Culex* vectors is dose dependent, the magnitude of serum viremia in a bird determines its host competence [1], [4], [6].

Resident birds are considered to be more important for the spread of WNV across the U.S. than migratory birds [7]. The house sparrow (HOSP; *Passer domesticus*) is a resident passerine and is highly competent for WNV [1], [5]. Additionally, HOSPs are ubiquitous across North America in urban, suburban, and rural landscapes and are a frequent bloodmeal source for *Culex* mosquitoes [8]–[10]. Unlike infection in American crows (*Corvus brachyrhynchos*), infected HOSPs sustain viral titers above the threshold required for mosquitoes to become infected but exhibit a low mortality rate [5]. However, reports have suggested that WNV causes enough mortality to contribute to a declining population of HOSPs in the U.S. [11]–[13]. The WNV seroprevalence of HOSPs has been estimated to fluctuate annually and locally [14], [15], with levels reaching as high as 40% during outbreak years [16].

Since the first identification of WNV in North America, the virus has diverged into 3 described genotypes. By 2003, the original East Coast genotype was replaced by the North American WN02 genotype, defined by a valine-to-alanine amino acid substitution at codon 159 in the envelope protein (E-V159A) [17], [18]. There are reports that suggest viral isolates containing this mutation may increase the rate of WNV dissemination in *Culex* mosquitoes [17], [19], [20]. A third genotype, SW03, was first described for WNV isolates collected in the southwest U.S. in 2003 [21]. The SW03 genotype is characterized by the E-V159A substitution in conjunction with an alanine-to-threonine substitution at codon 85 in the NS4A protein (NS4A-A85T). The NS4A-A85T mutation has not been specifically assessed for differential viral phenotypic effects in either avian hosts or mosquito vectors. Isolates obtained during routine surveillance since 2003 have largely been limited to WN02 and SW03 genotypes that have been found co-circulating in the U.S. as recently as 2012 [22], [23].

Dual-host viruses such as WNV have many constraints on viral evolution. Due to the necessity for replication in birds and mosquitoes for its enzootic maintenance in North America, WNV has been subject to widespread purifying selection [21], [23], [24] to maintain efficient replication in two disparate hosts [25], [26]. However, WNV has adaptively evolved at discrete loci during its 15 years of circulation in North America [21], [23], and whether or not this evolution has been driven by passerines is unknown. In order to assess the possibility that transmissibility of WNV could be a driving force for the fixation of the E-V159A substitution and alternative genotype-specific amino acid substitutions in North America, the competence of HOSPs for East Coast, WN02, and SW03 genotype viruses were compared. Furthermore, the competence of North American HOSPs for the same founding East Coast strain, NY99, was also evaluated over 14 years to identify potential co-evolutionary signatures in an avian host.

## Materials and Methods

### Sequence analyses of WNV isolates

A protein alignment of 132 WNV isolates was performed using Clustal Omega [27]. Twelve of these isolates were used for experimental inoculation of HOSPs in this study. A maximum likelihood phylogeny was constructed with 1,000 bootstrap replicates using PhyML [28]. Non-synonymous diversity and divergence calculations were performed using DNAsp v5 [29]. Sequences for isolates TX8759 and TX8779 were determined as described previously and have been assigned GenBank accession numbers KJ786936 and KJ786935, respectively [30].

### Collection and infection of wild HOSPs

Wild HOSPs were trapped in Larimer County, CO, in 2012–2013 using mist nets. Serum from each bird was tested for WNV neutralizing antibodies using a 90% plaque reduction neutralizing test as reported previously [31]. Groups of 5–8 seronegative birds were inoculated subcutaneously with 1,500 PFU of WNV. Blood was collected daily by jugular venipuncture for 7 days post-inoculation. Blood was immediately diluted 1∶10, coagulated for 30 minutes at room temperature, and spun for 10 minutes at 2500× g. Serum viral titers were quantified using Vero cell plaque assay as reported previously [32]. The lower limit of detection for this assay was 1.7 log_10_ PFU/mL.

### Reservoir competence index calculations

Reservoir competence was calculated as the product of HOSP susceptibility, mean daily HOSP infectiousness, and duration of infectiousness for mosquitoes, as previously reported [5]. A value of 1.0 for HOSP susceptibility was used for all WNV isolates, as 100% of challenged birds demonstrated viremias. The lower threshold of HOSP serum viremia considered infectious to mosquitoes was 4.7 log_10_ PFU/mL [4]. Infectiousness was calculated based on a linear regression analysis as the proportion of mosquitoes predicted to become infected after feeding on a host with known viremia [4], [6], [33], [34].

### Statistical analyses

Statistical significance of differences in peak viremia and reservoir competence was calculated using ANOVA. A Mantel-Cox log-rank test was used to compare survival curves. For regression analyses, r^2^ values were used to determine the best model, and a linear model was used. All calculations were performed using GraphPad Prism 6 (San Diego, CA) or R (www.R-project.org).

### Ethics statement

This work was performed under approved institutional animal care guidelines. Protocols were approved by the Institutional Animal Care and Use Committees at the Division of Vector-borne Diseases, Centers for Disease Control and Prevention (approval number 13-009), the University of California, Davis (approval numbers 12874 and 15895), and Colorado State University (approval number 10-2078A).

## Results

### Three major WNV genotypes in North America

**Figure 1:**
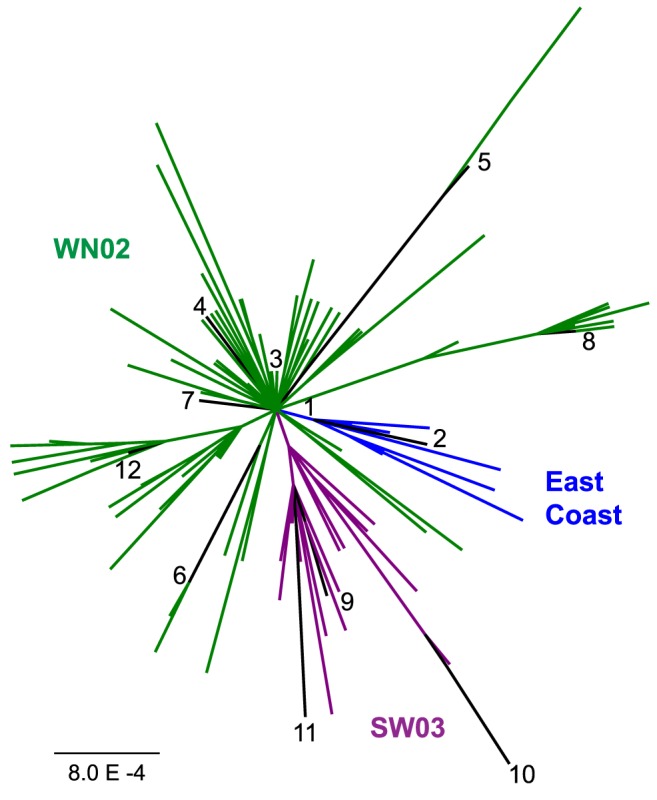
Maximum likelihood phylogeny of a protein alignment for 132 North American WNV isolates. East Coast viruses are blue; WN02 (E-159A) viruses are green; and SW03 (E-159A/NS4A-85T) viruses are purple. Viruses used in this study are numbered according to Table 1.

Previous studies of WNV evolution in North America have identified 3 major genotypes: East Coast, which includes the prototypic NY99 strain that was the first isolate sequenced during the U.S. epidemic but is no longer known to be in circulation; WN02, characterized by a valine-to-alanine mutation at E-159; and SW03, characterized by the E-V159A substitution and an alanine-to-threonine substitution at NS4A-85 (Table 1). These broad groups form three clusters in a phylogeny of North American isolates (Fig. 1). However, the SW03 genotype also includes some isolates that cluster within the WN02 genotype, such as isolate 12 (TX2689; Fig. 1). This suggests that the NS4A-A85T substitution has occurred independently on multiple occasions, and the SW03 genotype encompasses a group of viruses with variable genetic backgrounds.

**Table 1:** WNV isolates used in this study.

	Isolate	Location	Year	Species	Genbank accession	E-159	NS4A-85
*East Coast genotype*							
1	NY99	New York	1999	Chilean flamingo	AF196835	V	A
2	NY2001	New York	2001	Human	KJ786934	V	A
*WN02 genotype*							
3	TX114	Texas	2002	Blue jay	GU827998	A	A
4	TX7191	Texas	2007	Blue jay	JF415920	A	A
5	TXAR7465	Texas	2009	*Culex tarsalis*	JX015521	A	A
6	TX8759	Texas	2012	Blue jay	KJ786936	A	A
7	TX8779	Texas	2012	Northern mockingbird	KJ786935	A	A
8	TX2600	Texas	2012	*Cx. quinquefasciatus*	KC736487	A	A
*SW03 genotype*							
9	M19433	Texas	2009	*Aedes albopictus*	JF415919	A	T
10	TXAR6115	Juarez	2009	*Cx. quinquefasciatus*	JX015520	A	T
11	TXAR6572	Texas	2010	*Cx. tarsalis*	JX015523	A	T
12	TX2689	Texas	2012	*Cx. quinquefasciatus*	KC736492	A	T

In general, the East Coast genotype contains lower genetic diversity compared to the WN02 and SW03 groups (Table 2). Fewer East Coast genotype isolates are available because it was circulating for only a few years, compared to nearly a decade of circulation and diversification for WN02 and SW03 genotypes [35]. This is reflected in the WNV phylogeny, where the relative sizes of each genotypic population are emphasized, and in the proportions of the isolates we chose to test (Fig. 1). In order to examine the fitness effects of WNV diversity, we selected 12 isolates collected between 1999 and 2012 (Table 1) that recapitulate the genetic variation and divergence of WNV in the U.S (Tables 2–4). We chose two East Coast isolates from New York, six WN02 isolates from Texas, and four SW03 isolates from Texas and Mexico, identified in Figure 1 by numbers 1–12.

**Table 2:** Genetic diversity of North American WNV genotypes.

	nonsyonymous diversity*			nonsynonymous divergence*	
	this study	larger sample		this study	larger sample
East Coast	3.8 (n = 2)	6.9 (n = 11)	East Coast vs. WN02	8.7 (n = 8)	9.9 (n = 109)
WN02	11.0 (n = 6)	9.6 (n = 98)	East Coast vs. SW03	12.1 (n = 6)	11.9 (n = 30)
SW03	14.4 (n = 4)	10.4 (n = 19)	WN02 vs. SW03	14.0 (n = 10)	11.8 (n = 117)

*mean substitutions/site ×10^−4^.

**Table 3:** Amino acid differences between East Coast and WN02 genotype isolates.

	C			E							NS1		NS2A						NS2B			NS3			NS4B	NS5			
virus	104	119	121	51	89	123	159	253	332	396	236	308	26	52	89	90	95	188	99	119	121	162	188	334	14	49	314	647	860
NY99	K	A	V	A	A	T	**V**	I	T	H	I	I	K	T	F	M	L	R	M	V	V	I	Q	S	S	V	K	K	A
2	.	.	.	.	.	.	.	.	.	Y	.	.	.	.	.	V	.	.	.	.	.	.	K	.	.	.	.	.	.
3	.	.	.	.	.	.	**A**	.	.	.	.	.	.	.	.	.	.	.	.	.	.	.	.	.	.	.	.	.	.
4	.	.	.	.	.	.	**A**	.	.	.	.	.	.	.	.	.	.	.	T	.	.	M	.	.	.	.	.	.	T
5	.	V	A	T	.	.	**A**	.	M	.	.	.	R	.	L	.	.	.	.	L	.	.	.	.	.	.	R	.	.
6	R	.	.	.	.	.	**A**	.	.	.	V	.	.	.	.	.	.	K	.	.	I	.	.	.	.	I	.	.	.
7	.	.	.	.	.	.	**A**	V	.	.	.	.	.	.	.	.	.	.	.	.	.	.	.	.	.	.	.	R	.
8	.	S	.	.	V	N	**A**	.	.	.	.	V	.	I	.	.	F	.	.	.	.	.	.	T	I	.	.	.	.

**Table 4:** Amino acid differences between NY99 and SW03 genotype isolates.

	M	E			NS1	NS2A						NS2B	NS3			NS4A	NS4B		NS5					
virus	140	159	168	467	314	46	58	119	188	190	224	116	160	258	355	85	240	249	44	91	202	314	560	860
NY99	V	**V**	S	A	R	F	V	H	R	K	A	L	S	V	Y	**A**	I	E	R	M	Y	K	D	A
9	.	**A**	.	.	.	.	.	.	.	.	.	.	A	.	.	**T**	M	.	.	V	.	R	.	.
10	.	**A**	T	S	K	.	.	Y	.	.	V	M	.	I	F	**T**	.	.	.	.	F	.	E	.
11	A	**A**	.	.	.	L	.	.	.	R	.	.	.	.	.	**T**	.	G	K	.	.	R	.	T
12	.	**A**	.	.	.	.	I	.	K	.	.	.	.	.	.	**T**	M	.	.	.	.	.	.	.

### Effect of WNV evolution on viral replication profiles in contemporary HOSPs

**Figure 2:**
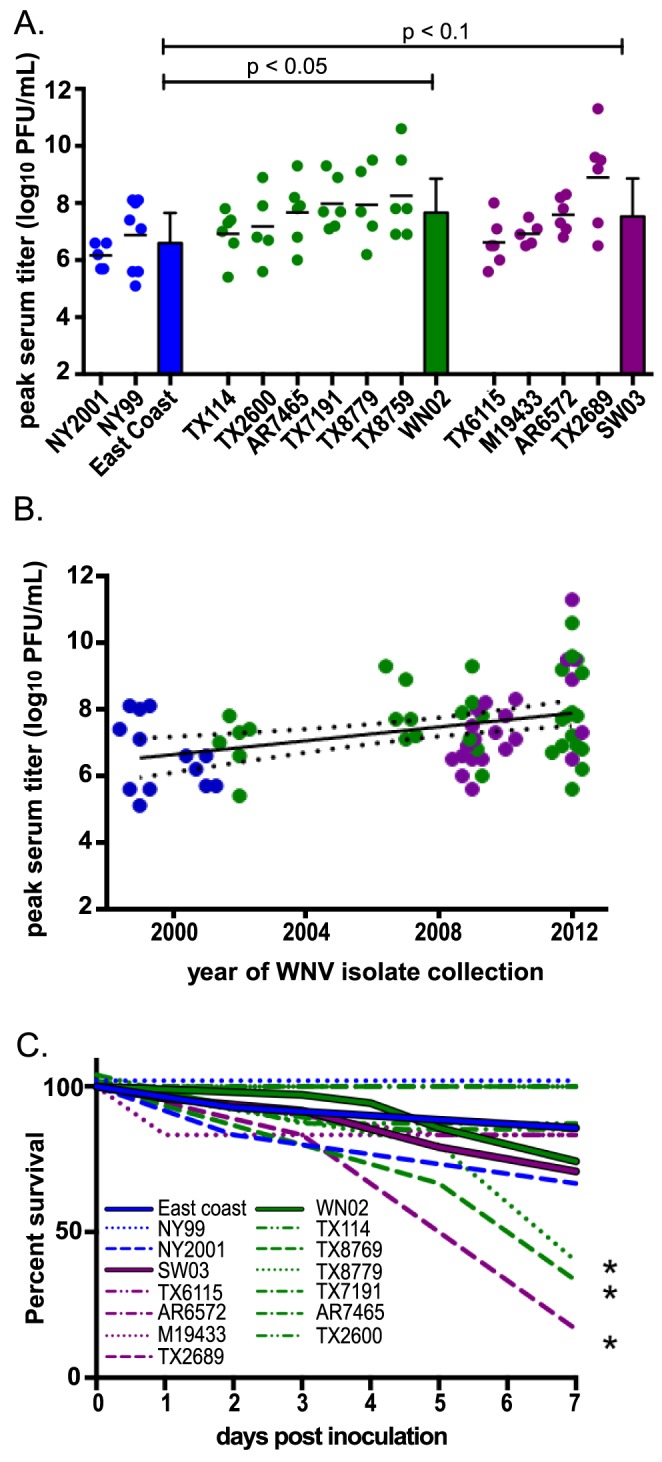
Peak viremias and mortality in HOSPs by viral isolate and over time. Points represent individual birds. HOSPs inoculated with East Coast viruses are designated in blue; HOSPs inoculated with WN02 viruses are designated in green; and HOSPs inoculated with SW03 viruses are designated in purple. (A) Peak serum titers for individual HOSPs inoculated with one of the 12 WNV isolates. Bars represent the mean peak titer for each WNV genotype. Error bars reflect the standard deviation of the mean. (B) Linear regression analysis of peak serum titer for individual birds inoculated with WNV, stratified by year of virus collection. 95% confidence intervals are shown by the dotted lines. (C) Percent HOSP survival for 7 days post-inoculation. Viral isolates are represented by dashed lines, and genotype means are represented by solid lines. *p<0.05.

To determine whether the WN02 displacement of the East Coast genotype was the result of viral adaptation to North American avian hosts, groups of HOSPs collected in 2012 and 2013 were inoculated with 12 WNV isolates representing the three North American genotypes: East Coast, WN02, and SW03 (Table 1). In total, seventy-two birds were collected and inoculated with WNV in 2012–2013, and viremias were measured daily for 7 days. As expected for wild-caught birds, there was considerable variability in viral titers among replicates within groups (Fig. 2a). While the peak viral titer was generally observed on day 3 for HOSPs inoculated with any WNV genotype, peak viremias of individual birds occurred on different days. To determine the overall peak titer for each virus, the peak viral titer for individual HOSPs was determined irrespective of the day post-inoculation and then averaged. The peak titers also were averaged by viral genotype, and peak viral titer varied significantly by genotype. WN02 viruses induced a mean peak titer in HOSPs that was 10-fold greater than East Coast viruses (Fig. 2a, p<0.05). SW03 viruses produced a similar 10-fold increase in mean peak viral titer over East Coast viruses, though this difference was not significant (p = 0.09). This is likely due to the large amount of variation in viral titers observed from inoculated HOSPs (Fig. 2a).

To investigate whether viral adaptation to HOSPs has occurred over time, peak viral titers were analyzed by year of viral isolate collection. Linear regression analysis indicated that peak viral titer increased at a significant rate (Fig. 2b, p<0.05) with an average increase in peak titer of 0.10 log_10_ PFU/mL sera per year (95% CI: 0.04 to 0.16). The mean peak viral titer induced in HOSPs collected in 2012–2013 by WN02 and SW03 viruses isolated in 2012 was 1.2 log_10_ PFU/mL sera higher than the peak titer generated by NY99 in HOSPs collected in 2012–2013. This analysis is consistent with the corresponding chronologic appearance of East Coast vs. WN02/SW03 genotypes and indicates that WNV has adapted to HOSPs over time.

Moderate mortality is characteristic of WNV infection in the HOSP. The percentage of surviving birds was calculated for each virus for 7 days post-infection. On average, HOSPs collected in 2012 and inoculated with East Coast, WN02, and SW03 isolates had similar survival curves, with a mean mortality of 15–30% by 7 days post-infection (Fig. 2c). However, three viruses had significantly different survival profiles: TX2689 (SW03), TX8759 (WN02), and TX8779 (WN02), which resulted in 65–85% HOSP mortality by 7 days post-infection (p<0.05) and induced the highest peak viral titers among the 12 WNV strains tested in HOSPs (8.9, 8.3, and 7.9 log_10_ PFU/mL sera, respectively; Fig. 2a). Interestingly, these 3 viruses were isolated in 2012, indicating that some WNV strains circulating in 2012 may have been more pathogenic to HOSPs than those isolated in previous years. Significantly, the two viruses with the highest mortality and peak viral titers (TX2689 and TX8759; Fig. 2b and 2c) share a common amino acid substitution, NS2A-R188K (Tables 3 and 4), that emerged in North America as early as 2008 [23].

### Variability in HOSP competence for WNV isolates

**Figure 3:**
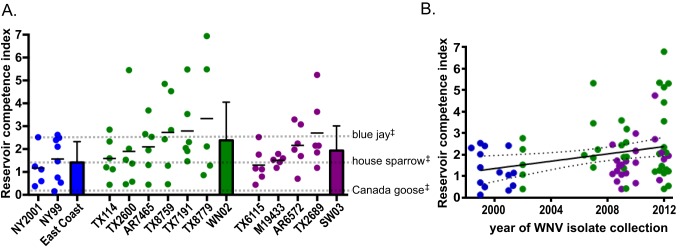
HOSP reservoir competence for WNV. Points represent individual birds. Birds inoculated with East Coast viruses are designated in blue; birds inoculated with WN02 viruses are green; and birds inoculated with SW03 viruses are purple. (A) Reservoir competence index calculated for individual birds inoculated with one of the 12 WNV isolates, and the mean competence for each WNV genotype. ^‡^Previous calculations of reservoir competence indices for blue jay, HOSP, and Canada goose with NY99 [5] are shown in dashed lines for comparison. (B) Linear regression analysis of reservoir competence for individual birds inoculated with WNV, stratified by year of virus collection. 95% confidence intervals are shown by dotted lines.

Infectiousness of WNV-infected HOSPs for mosquitoes is a combination of both the magnitude and duration of viremia. In the absence of performing vector competence studies for all of the viruses assessed, the mean reservoir competence index for each viral isolate in HOSPs was generalized by predicting the proportion of mosquitoes likely to become infected using linear regression analysis based on previously published data [4], [6], [33], [34]. With these calculations, an index value of 1.0 would indicate that 100% of mosquitoes feeding on a host for 1 day would be predicted to become infected by the host, though it does not predict the number of mosquitoes that would transmit WNV or the effects on mosquito survival. The mean competence index for HOSPs infected with WNV isolates from the WN02 genotype was 2.4, compared to 1.1 for the East Coast genotype (Fig. 3a), indicating that 120% more mosquitoes would be predicted to become infected after feeding on HOSPs infected with a WN02 isolate compared to mosquitoes feeding on HOSPs infected with an East Coast isolate. The mean HOSP competence index for the SW03 genotype was 1.9 (Fig. 3a), meaning that 73% more mosquitoes would become infected after feeding on HOSPs infected with a SW03 isolate than mosquitoes feeding on HOSPs infected with an East Coast isolate, and 26% more mosquitoes would become infected from feeding on HOSPs infected with a WN02 isolate compared to feeding on HOSPs infected with a SW03 isolate. These results were compared to previously published WNV competence indices for birds inoculated with the NY99 strain of WNV. Species of the avian order Anseriformes, such as the Canada goose (*Branta canadensis*), have WNV competence indices close to 0 and are considered non-competent hosts [5], [6]. Estimates for passerines suggest competence indices of at least 1, with HOSPs having values between 1 and 1.5, and members of the *Corvidae* family, such as the blue jay (*Cyanocitta cristata*), having values between 1.5 and 2.5 [5], [6]. HOSPs inoculated with 6 of the 12 tested WNV isolates, including NY99, had competence indices within the typical range of HOSPs (NY99, NY2001, TX114, TX2600, TX6115, M19433; Fig. 3a). However, HOSPs inoculated with 6 other isolates had competence indices greater than 2, which is more similar to the range for corvids (AR7465, TX8759, TX7191, TX8779, AR6572, TX2689; Fig. 3a). When the reservoir competence indices were stratified by year of viral isolate collection, a significant association between year and index value was observed (Fig. 3b, p<0.05) with an average increase in reservoir competence of 0.09 per year (95% CI: 0.05 to 0.15). The WNV competence of HOSPs trapped in 2012–2013 for viruses collected over 13 years varied between 1.1 and 2.6, or a 140% increase in predicted mosquito infectivity.

### Change in HOSP modulation of WNV viremia during North American epizootic

**Figure 4:**
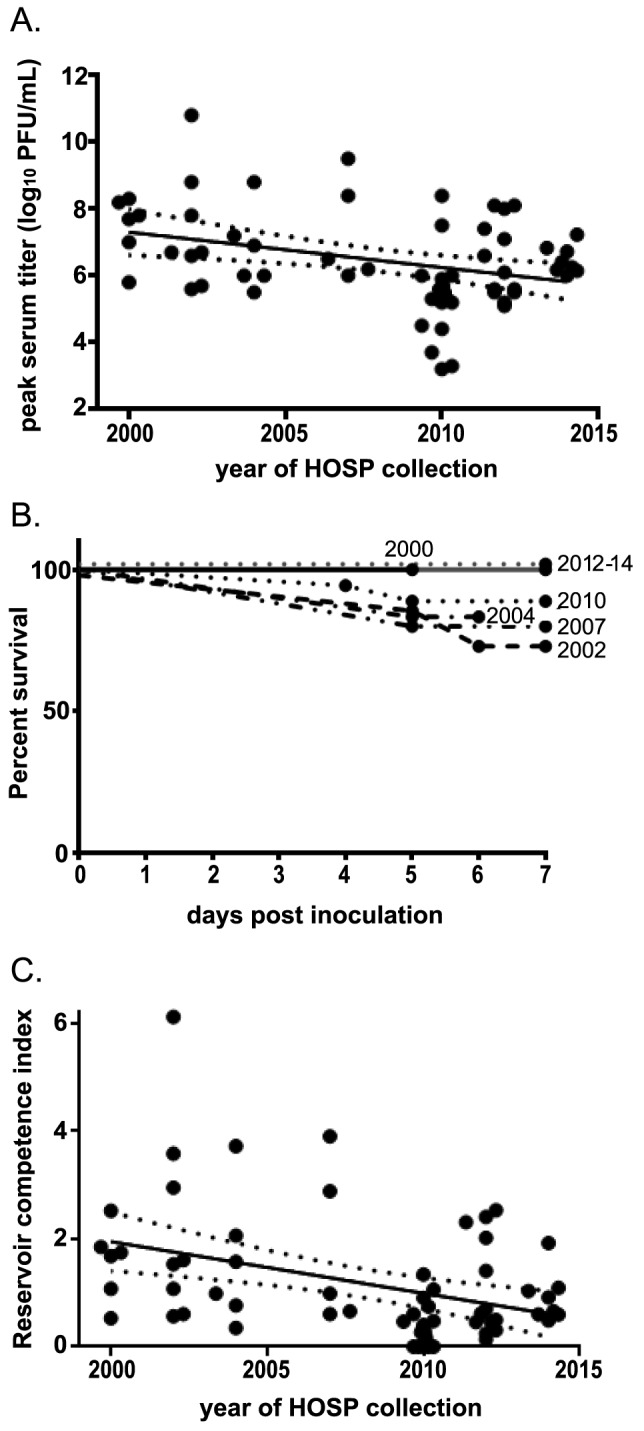
Peak viremias and mortality induced by the NY99 strain of WNV in HOSPs collected over time. Points represent individual birds. (A) Linear regression analysis of peak serum titer for individual birds inoculated with NY99, stratified by year of HOSP collection. Data from 2000 [5], 2002 [31], and 2004 [1] were previously published. (B) Percent HOSP survival for up to 7 days post-inoculation. (C) Linear regression analysis of reservoir competence for individual birds inoculated with NY99, stratified by year of HOSP collection. 95% confidence intervals are shown by dotted lines.

Given that WNV induces mortality in HOSPs and that mortality is likely related to the magnitude of viremia induced [36], it is likely that WNV infection has imposed a selective pressure on HOSPs for reduced infection-related mortality by reducing peak viremias. To determine whether HOSPs have modulated their ability to sustain WNV replication over time, results from similar experimental inoculations with the NY99 strain in HOSPs trapped between 2000 and 2014 were compared to data from this study using HOSPs trapped in 2012–2013. Peak viral titers were calculated for individual birds in 7 previous experiments, including 3 published studies [1], [5], [31], and analyzed by year of HOSP collection. Four of the previous experiments were performed at the Centers for Disease Control and Prevention using HOSPs trapped in Larimer County, CO, and 3 experiments were performed at the University of California-Davis using HOSPs trapped in Kern County, CA. These 2 geographically distinct populations of HOSPs showed no difference in peak viral titer over time; therefore, they were analyzed together. Using combined data from HOSPs trapped between 2000 and 2014, the peak viral titer for infected HOSPs was found to be significantly negatively associated with year of HOSP collection (Fig. 4a, p<0.05) with an average decrease in peak titer of 0.11 log_10_ PFU/mL sera per year (95% CI: 0.03 to 0.18). Overall, the mean peak viremia elicited by the NY99 strain in HOSPs has decreased by 1.0 log_10_ PFU/mL sera from 2000 to 2014. As expected, the 7-day survival of HOSPs inoculated with NY99 has increased from 75% in 2002 to 100% in recent years, though this trend is not significant (Fig. 4b). Accordingly, the mean host competency index also demonstrated a negative correlation with the year of HOSP collection (Fig. 4c, p<0.05) with an average decrease in reservoir competence of 0.10 per year (95% CI: 0.04 to 0.15). The mean competence index value for HOSPs inoculated with NY99 has decreased from 1.6 in 2000 to an estimated 0.9 in 2014. This difference would be expected to correlate with a decrease in mosquito infection of 44% for HOSPs inoculated with NY99.

## Discussion

WNV has evolved to replicate to higher peak titers in HOSPs (Fig. 2) since WNV emergence in North America in the late 1990s. Conversely, the founding East Coast genotype (NY99) has demonstrated a reduced capacity for eliciting infectious titers in HOSPs over time (Fig. 4). Taken together, these observations indicate a cyclic pattern of adaptive selection acting on WNV and avian hosts, suggestive of the ‘Red Queen’ hypothesis of evolution [37]. Mortality and fitness effects of high replication of the founding strain of WNV in HOSPs in North America may have served as a significant selective pressure for increased control of WNV replication in HOSPs that, in turn, may have selected for viral adaptations to increase viremia and therefore transmissibility to mosquitoes. Ultimately, because HOSPs inoculated with WNV only have decreased viremias in response to East Coast viruses that are no longer circulating, the consequence of viral evolution has been an increase in reservoir competence of HOSPs from 1.6 in 2000 to 2.6 in 2013 for extant WNV genotypes (Fig. 3). The Red Queen hypothesis would predict that HOSPs will further adapt to sustain lower viremias in response to WN02 and SW03 genotypes, with corresponding viral mutations selected in order to offset avian antiviral effects.

The variation in titers that we observed in HOSPs may be unrelated to selective pressures acting on the virus and host. However, WNV has been a significant cause of death for HOSPs in the U.S., and HOSP abundance has decreased in response to WNV infection [16]. Overall, HOSP abundance in the rural U.S. has decreased significantly by an average of 3.1% per year from 1999 to 2012 (p<0.05) [38]. The proportion of population decrease that is due to WNV infection is unknown, but the results from this study suggest WNV may have contributed to population decline recently due to higher WNV-induced mortality. Accordingly, selection acting on HOSPs to mediate lower WNV-induced mortality is a plausible explanation. One potential mechanism of increased survival in HOSPs is a better regulation of viral titers, modulated by a change in the innate immune response to WNV. As WNV is known to antagonize the host interferon response [39]–[44], the host may be able to modulate viral titers by evasion of viral antagonism. Sequencing of HOSP innate immune genes from archival samples may reveal genetic differences between individuals demonstrating variable viremias and mortality profiles.

Based on the subsequent increase in viremias induced by contemporary WNV strains in HOSPs, it is possible that HOSPs have exerted a selective pressure on WNV that contributed to the emergence of the WN02 and SW03 genotypes. The observed increase in viremia in HOSPs also may be the consequence of a general viral adaptation to mosquitoes, multiple avian species, or a specific viral adaptation to another avian species in North America, such as the American robin (AMRO; *Turdus migratorius*), that is thought to be the most preferred host for mosquito bloodmeals [45]. To test whether AMROs may have driven the evolution of WN02 and SW03 genotypes, similar experimental inoculations with viral isolates collected during different years would need to be performed. However, it is unlikely that WNV has adapted to American crows (AMCRs), as all North American WNV strains are uniformly pathogenic to AMCRs due to the conserved proline at NS3-249 in North American isolates [46]. Although pathogens are generally assumed to evolve towards decreased pathogenicity in a susceptible host, there are examples of short-term increases in pathogen virulence in birds, such as the emerging bacterium *Mycoplasma gallisepticum* in wild house finches (*Haemorhous mexicanus*) [47]. For example, higher host mortality, which increases mosquito transmissibility of WNV, may increase viral spread by reducing flock immunity [48].

HOSPs were introduced into North America in the 1850s [49]. Thus, divergence between New and Old World HOSP populations is likely, and these experiments with North American HOSPs may not be consistent with other geographically distinct HOSP populations. Interestingly, amino acid variation at the E-159 locus has been observed in Old World Lineage 1A WNV isolates several times prior to the introduction of WNV to North America [50], as well as Lineage 2 WNV strains [51], but no other lineage has acquired an alanine at this position. Since HOSPs are prevalent in Europe and Africa and would likely serve as important avian hosts, it is possible that other substitutions at E-159 are beneficial to viral replication in distinct HOSP populations. The NS4A-85 locus is also hyper-variable among WNV strains (Table 4), with a threonine, valine, or isoleucine present in other Lineage 1 and 2 viruses. The NS2A-R188K mutation that was associated with higher peak viral titers and mortality in inoculated HOSPs presented herein (Fig. 2, Tables 3–4) is also found in Lineage 2 viruses. Although statistical analyses of WNV evolution do not identify these sites as the targets of diversifying selection, the variability of these sites across lineages combined with observed phenotypic effects in HOSPs suggests they may be adaptive changes.

The evolution of WNV strains that have increased the magnitude and duration of viremias in HOSPs highlights the potential importance of HOSPs for the maintenance of WNV in North America. Furthermore, declining WNV-induced mortality in HOSPs infected with an early WNV strain suggests that WNV has imparted a significant selective pressure on wild bird populations. This evidence of virus-host co-evolution suggests that the competence of North American birds for WNV is likely to continue to change.
